# Influence of Coupled Activated Recycled Fine Powder on the Performance of Ultra-High-Performance Concrete

**DOI:** 10.3390/ma19010201

**Published:** 2026-01-05

**Authors:** Chun Lu, Ming Zhang, Nirmal Shrestha, Dongdong Yang, Chengxiao Yu

**Affiliations:** School of Civil Engineering and Transportation, Yangzhou University, Yangzhou 225127, China; 19515796709@163.com (C.L.); nirmalshrestha403@gmail.com (N.S.); ddyang@yzu.edu.cn (D.Y.); 15651816032@163.com (C.Y.)

**Keywords:** calcination, compressive strength, grinding, activation, recycled fine powder, ultra-high-performance concrete

## Abstract

Ultra-High-Performance Concrete (UHPC) is being increasingly utilized in major engineering projects due to its excellent mechanical properties, strong durability, and superior overall performance. Nevertheless, the widespread use of premium cementitious materials leads to high expenses and a substantial environmental impact. In this work, crushed recycled paste was calcined at 600 °C for two hours to produce calcined recycled fine powder (RFP) with varying hydration reactivity. UHPC was produced using the RFP in place of some of the cement. Chemical activation was accomplished by adding a composite activator system made up of Ca(OH)_2_, Na_2_SO_4_, Na_2_SiO_3_·9H_2_O, and K_2_SO_4_ in order to further improve the performance of UHPC. Particle size, viscosity, fluidity, mechanical properties, and hydration products were analyzed to establish the best activator type and dosage, as well as the ideal activation procedure for recycled fine powder. By mass replacement of cementitious materials, when 15.0% of the calcined recycled fine powder was added, the compressive strength of UHPC reached 149.1 MPa, a 23.2% increase over reference UHPC without calcined recycled fine powder. The results show that the calcined recycled fine powder ground for 60 min exhibits the highest activity. More hydrated products were formed in UHPC as a result of the addition of Ca(OH)_2_. The compressive strength peaked at 162.2 MPa at an incorporation rate of 1.5%, which is 8.8% higher than UHPC without an activator.

## 1. Introduction

China’s increased infrastructure development has produced a significant amount of waste concrete. The solidified mortar of the leftover concrete is ground to produce the recycled fine powder [1]. Recycled fine powder has some potential reactivity because it contains a considerable number of hydrated products and a specific percentage of un-hydrated cement particles [2,3,4,5,6]. The potential reactivity of recycled fine powder can be successfully triggered to some degree through procedures like chemical activation, mechanical grinding, and heat treatment.

The activation effects of thermal treatment, chemical activation, and their combination of recycled fine powder were examined by Tian Qing et al. [7]. Their research showed that while calcination modifies the mineral structure of the original components in the powder, chemical activation produces an alkaline environment and reactive ions. It was demonstrated that both techniques may give the recycled fine powder hydraulic action. Kang Xiaoming et al. [8] examined the activation effects on recycled fine powder using a variety of techniques, such as thermal treatment at 600 °C and 800 °C, and the activators Ca(OH)_2_ and Na_2_SiO_3_·9H_2_O. According to the study, heat activation at 800 °C produced the best results, followed by chemical activation with Ca(OH)_2_. The ideal temperature for activating recycled fine powder through calcination is 600 °C, according to research by Liu Yanni et al. [9]. The effects of combining chemical and physical activation techniques on the reactivity of recycled fine powder were examined by Xiong Kexing et al. [10]. The study showed that this method was considerably more effective than the single activation method, increasing the reactivity index of recycled fine powder by 14.7%.

UHPC has drawn a lot of attention and widespread use in recent years due to its exceptional qualities, which include great strength and durability. However, a lot of cementitious materials, including cement and ultra-fine powders, are used in the manufacturing of UHPC [11,12]. As a result, problems like high cost and significant environmental impact prevent its widespread use. Thus, it is crucial for its application to develop low-carbon UHPC preparation methods without substantially compromising important features [13,14,15]. In accordance with China’s “dual carbon” rules, the activation treatment of recycled fine powder and its application in the creation of UHPC not only increases the resource utilization level of waste concrete but also lowers the amount of cement utilized.

The impacts of composite fine powder and aeolian sand on the mechanical characteristics of UHPC were examined by Liu Chao et al. [16]. According to their research, the best mechanical qualities of UHPC were obtained by substituting 10.0% of cement with composite fine powder and 30.0% of river sand with aeolian sand. The impact of Ca(OH)_2_ on the UHPC using recycled and waste tuff fine powder was examined by Daosheng Sun et al. [17]. Their findings showed that activating with Ca(OH)_2_ increased the reactivity of both recovered fine powder and discarded tuff fine powder. Alkali-activated UHPC was created by X.Y. Zhang et al. [18] by replacing the slag in cementitious materials with recycled fine powder. The findings showed that when the water-to-binder ratio was between 0.27 and 0.29 and the recycled fine powder content was between 10.0% and 30.0%, the UHPC demonstrated good rheological and toughened properties.

The activation of recycled fine powder was restricted to regular concrete in earlier research. The amount of recycled fine powder that could be substituted for cement was not maximized since the activation of the recycled fine powder in UHPC was either not taken into consideration or only one activation method was used. Previous studies have identified 600 °C as the optimal calcination temperature [19], which aligns with findings reported by other researchers. This study uses recycled fine powder that was activated by calcination at 600 °C for two hours and then mechanically ground for use in UHPC preparation in order to overcome the problem of low reactivity in recycled fine powder. Chemical activators were also used to improve UHPC’s characteristics. Microstructural testing was used to examine the differences in hydration products of the resulting UHPC.

## 2. Materials and Methods

### 2.1. Materials

**Figure 1:**
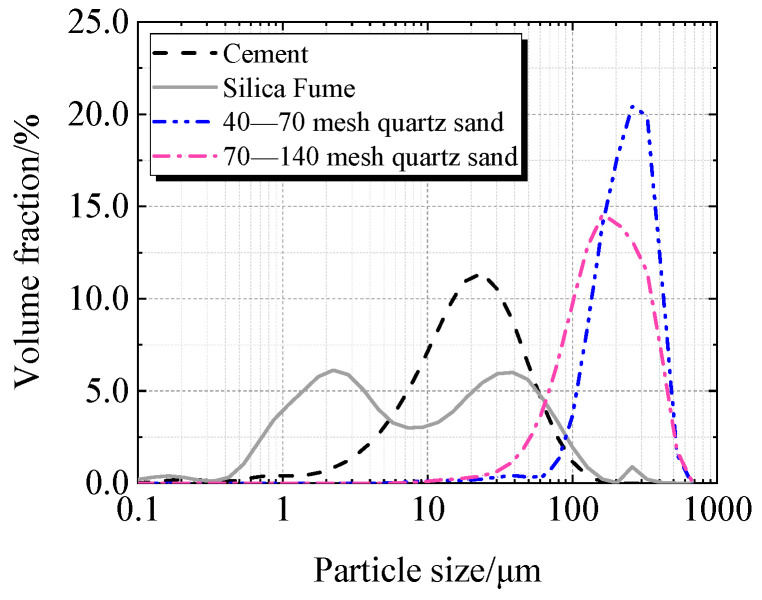
Particle size distribution of raw material.

**Figure 2:**
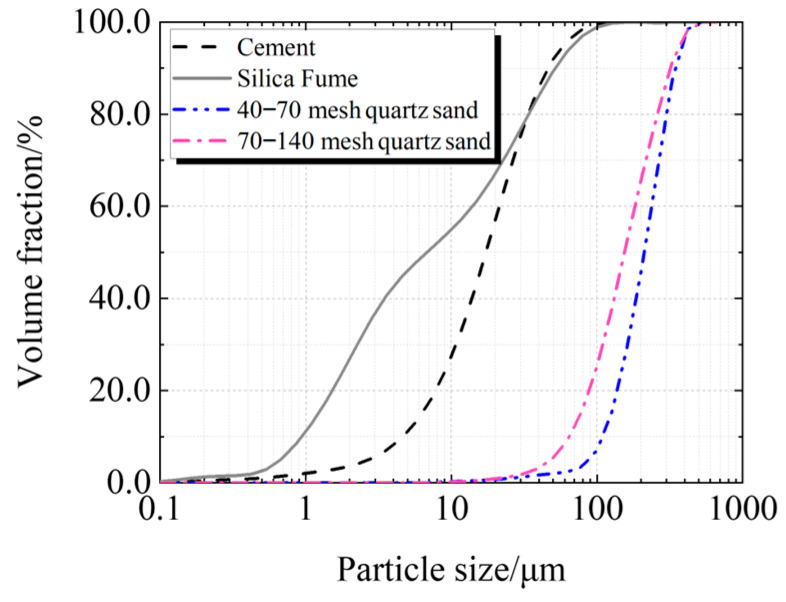
Cumulative particle size distribution of raw material.

Cement, silica fume, quartz sand, and superplasticizer are the primary components of UHPC. Taizhou Yangwan Hailuo Cement Co., Ltd., Taizhou, China, is the supplier of P·II 52.5 Portland cement. The Lingshou Tuyun Mineral Products Processing Plant in Shijiazhuang, China, provided silica fume with a SiO_2_ level of 98.0%. Fengyang Shengli Quartz Sand Co., Ltd., Chuzhou, China, provided two sizes of quartz sand: 425 μm to 212 μm and 212 μm to 106 μm. Polycarboxylate super-plasticizer from Sobute New Materials Co., Ltd., Nanjing, China, has a solid content of 51.5%. Figure 1 and Figure 2 display the particle size distributions of quartz sand, silica fume, and cement.

**Figure 3:**
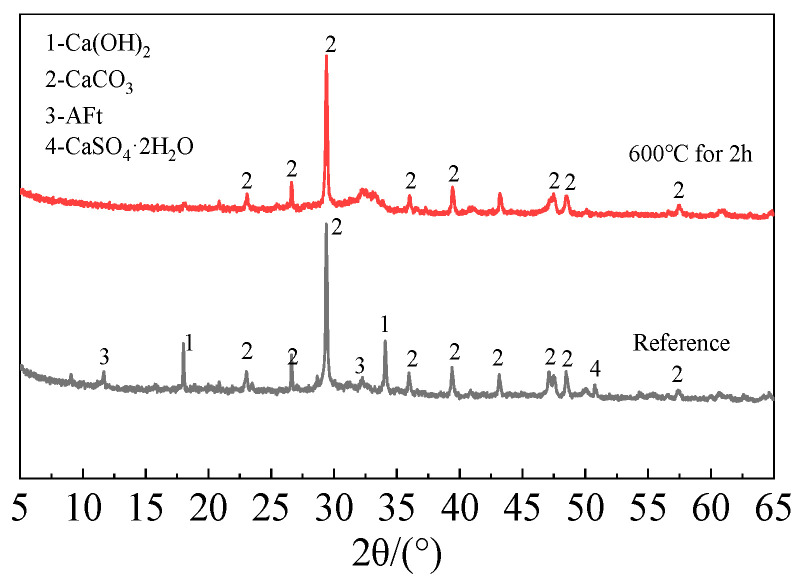
XRD pattern of recycled fine powder.

**Figure 4:**
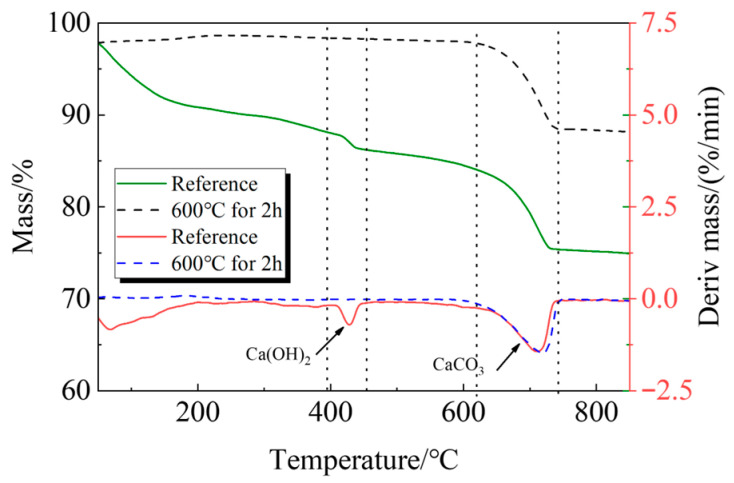
TG-DTG of recycled fine powder.

The following process was used to create the recycled fine powder: First, long-term outdoor natural curing was applied to cement paste blocks with a water-to-cement ratio of 0.5. In order to obtain coarse recycled particles, these solid cement paste blocks were crushed before the experiment and sieved through a sieve with a 1 mm size. After that, the coarse recycled particle was heated at a rate of 10 °C per minute until it reached 600 °C in a high-temperature furnace (SX2-12-12A)(Shengwang Instrument Factory, Shaoxing, China). It was then kept at that temperature for 120 min, and after the power was turned off, it was allowed to cool gradually for 6 h to room temperature within the furnace. In order to produce calcined recycled fine powders of various fineness, the recycled powder was ultimately ground for 30, 60, 90, and 120 min, respectively. Figure 3 and Figure 4 display the XRD and TG-DTG of recycled fine powder with and without calcination (reference specimen).

All of the chemical activators, Ca(OH)_2_, Na_2_SO_4_, Na_2_SiO_3_·9H_2_O, and K_2_SO_4_, are analytical reagents. Sinopharm Chemical Reagent Co., Ltd., Shanghai, China, supplied Ca(OH)_2_ and Na_2_SO_4_. Tianjin Kemiou Chemical Reagent Co., Ltd., located in Tianjin, China, provided the Na_2_SiO_3_·9H_2_O and K_2_SO_4_.

### 2.2. Mix Proportion and Preparation Process

The fundamental mix proportion of UHPC was determined through single-factor experiments. An initial mix proportion was determined based on previous research, with the following composition: mass:cement:silica fume:quartz:sand:water:superplasticizer = 45.0%:5.0%:50.0%:10.0%:0.5% (a 50:50 blend of quartz sand with particle sizes of 425–212 μm and 212–106 μm). Subsequently, the silica fume content was varied by replacing cement in different proportions to evaluate the flowability and compressive strength of UHPC. The optimal silica fume content was determined to be 12.5%, which provided satisfactory fluidity and the highest compressive strength. Following this, while maintaining the silica fume content at 12.5%, the quartz sand content was varied to assess its influence on fluidity and compressive strength, leading to the identification of the optimal quartz sand content. This stepwise approach, where each subsequent raw material’s optimal content was determined based on previously established optimal contents of other materials, was systematically applied, ultimately yielding the fundamental UHPC mix proportion presented in Table 1. Depending on the composition, 5.0%, 10.0%, 15.0%, and 20.0% of the cement was replaced with recycled fine powder that had been processed for varying amounts of time [20]. Doses of 0.5%, 1.0%, 1.5%, and 2.0% of the total mass of binder were used to introduce chemical activators.

The following stages were taken in the UHPC preparation process: All of the cementitious materials and quartz sands were added to the mixer and combined for one minute. Next, half of the mass of the water and chemical admixture solution was added and mixed for five minutes. Finally, the residue solution was added to the mixer and stirred for an additional five minutes before being discarded.

### 2.3. Test Methods

The following steps were included in the mechanical grinding process: Fill a ball mill (type: WZM-5L) with 500 g of recycled fine powder, the ceramic grinding balls occupied one-third of the mill jar’s volume, set the grinding time, at a rotational speed of 100 r/min, run it for the specified amount of time, and then gather the sample for testing. An intelligent laser particle size analyzer (type: Bettersize 2000) (Bettersize Instruments Ltd., Dandong, China.) was used to examine the particle size distribution of recycled fine powder with varying grinding times.

The study examined the compressive strength of hardened UHPC as well as the fluidity and viscosity of fresh UHPC. Fresh UHPC was evaluated for fluidity in accordance with GB 2419-2005 [21].

The following steps were involved in the viscosity measuring procedure: The fresh UHPC was poured into a 500 mL beaker and placed under a digital viscometer (Type: DV-2) (Shanghai Fangrui Instrument Co., Ltd., Shanghai, China). The viscometer’s rotor was submerged in the fresh UHPC to a predetermined depth, and the viscosity value was recorded every minute after the viscometer ran. The final result was the average of three consecutive measurements.

The toughened UHPC was subjected to a strength test in compliance with GB/T 17671-2021 [22]. Before being demolded, prism samples measuring 40 mm by 40 mm by 160 mm were created and kept in a conventional curing room for a day. After that, the samples were steam cured at 90 °C for 48 h, and their compressive strength was measured once they had cooled to room temperature [23].

The conductivity and pH tests were conducted with reference to the literature [24], using a conductivity and pH meter (model HI1131). The procedure involved filling a 500 mL beaker with 500 g of distilled water and a magnetic stir bar. After that, the beaker was placed on a water bath with a magnetic thermostat. The thermometer, pH electrode, and conductivity electrode were all submerged in the beaker. The conductivity and pH meter were turned on after 10.0 g of the test powder was quickly added to the beaker, after the water temperature reached 25 °C. Up until the test was finished after 24 h, the conductivity and pH meter gathered data every 10 s.

The dried UHPC paste samples were crushed, powdered, and run through an 80 μm-opening sieve for the TG-DTG and X-ray diffraction tests. A D8 Advance polycrystalline X-ray diffractometer from the German company Bruker AXS (Karlsruhe, Germany) was used for the X-ray diffraction test. Diffraction patterns were recorded between 5° and 65° at a step size of 5°/min. A PerkinElmer Pyris 1 TGA from Springfield, IL, USA was used to perform the TG-DTG test in a nitrogen environment at 10 °C per minute to 850 °C.

## 3. Results and Discussion

### 3.1. Effect of Grinding Time on Properties of UHPC Mixed Recycled Fine Powder

**Figure 5:**
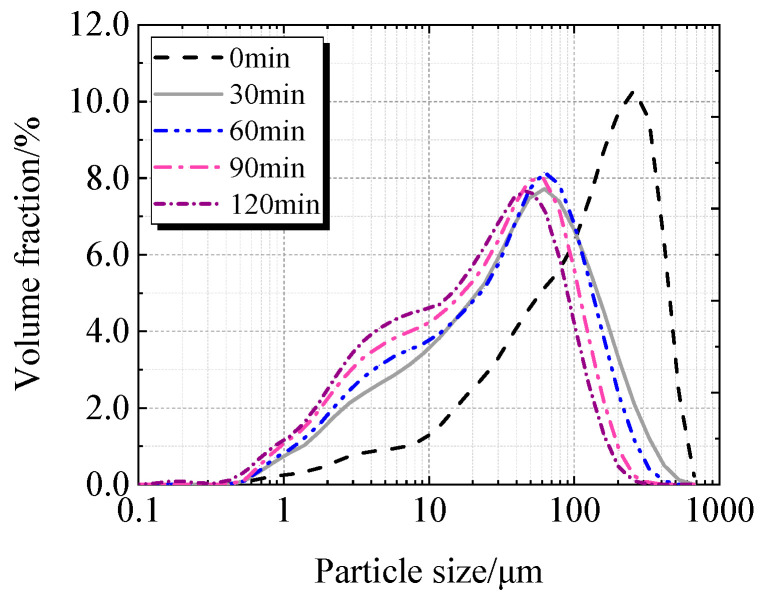
Particle size distribution of calcined recycled fine powder ground for different times.

**Figure 6:**
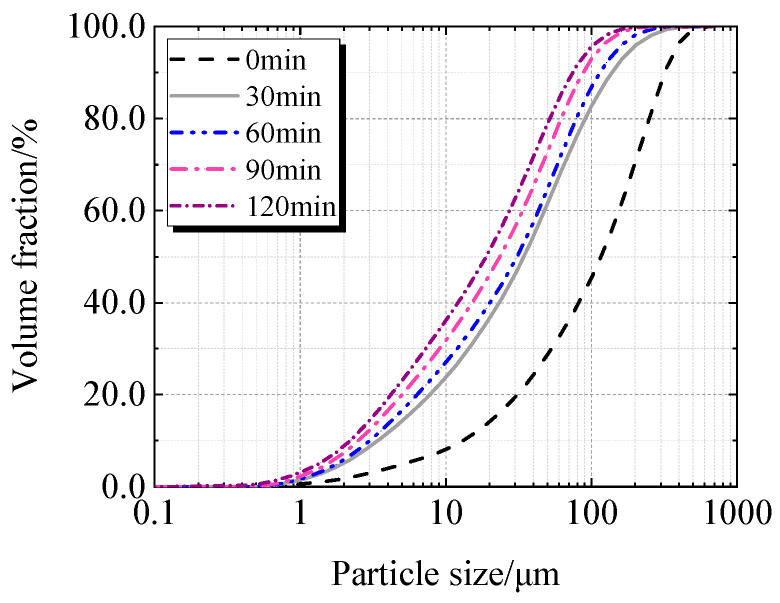
Cumulative particle size distribution of calcined recycled fine powder ground for different times.

Table 2, Figure 5 and Figure 6 display the particle characteristics of calcined recycled fine powder milled for various times. Table 2 shows that the calcined recycled fine powder’s particle size gradually decreases as the grinding time increases. The D_10_, D_50_, and D_90_ decrease when the grinding time exceeds 30 min, which is more noticeable when the grinding time exceeds 60 min. Figure 5 and Figure 6 show that the proportion of particles sized between 1 μm to 100 μm increases significantly with different grinding times.

**Figure 7:**
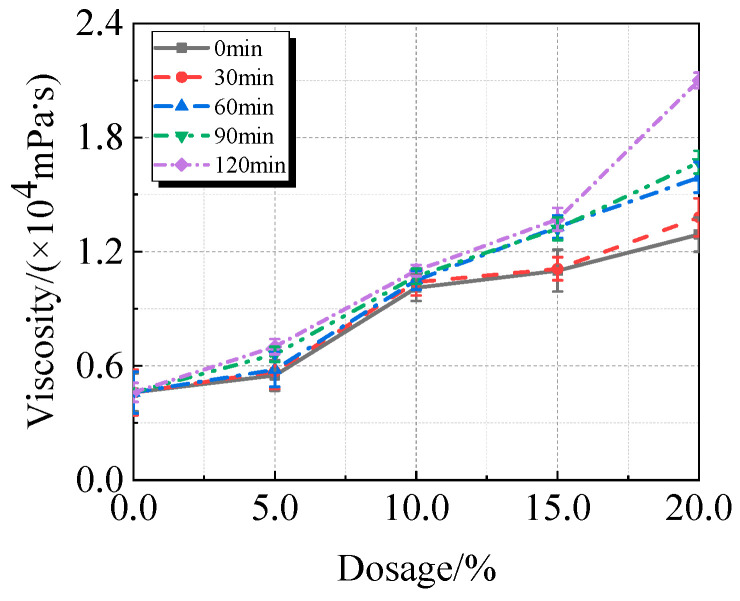
Viscosity of UHPC mixed with calcined recycled fine powder ground for different durations.

**Figure 8:**
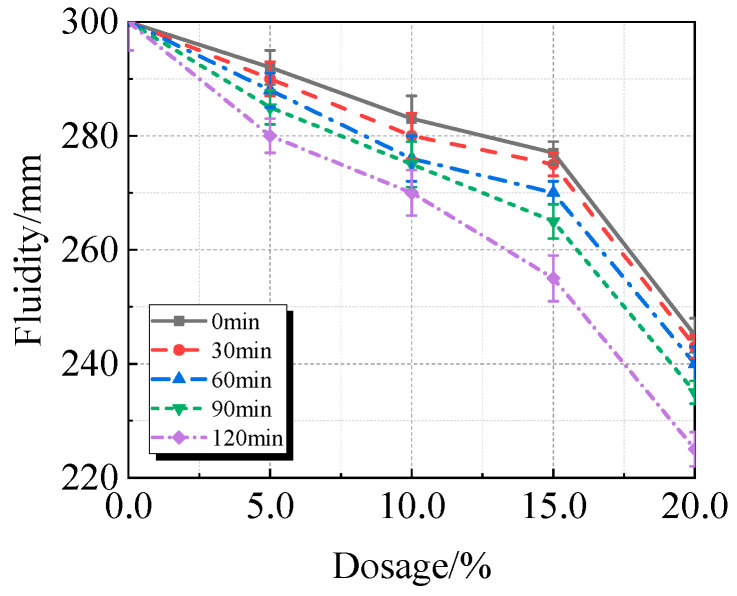
Fluidity of UHPC mixed with calcined recycled fine powder ground for different durations.

**Figure 9:**
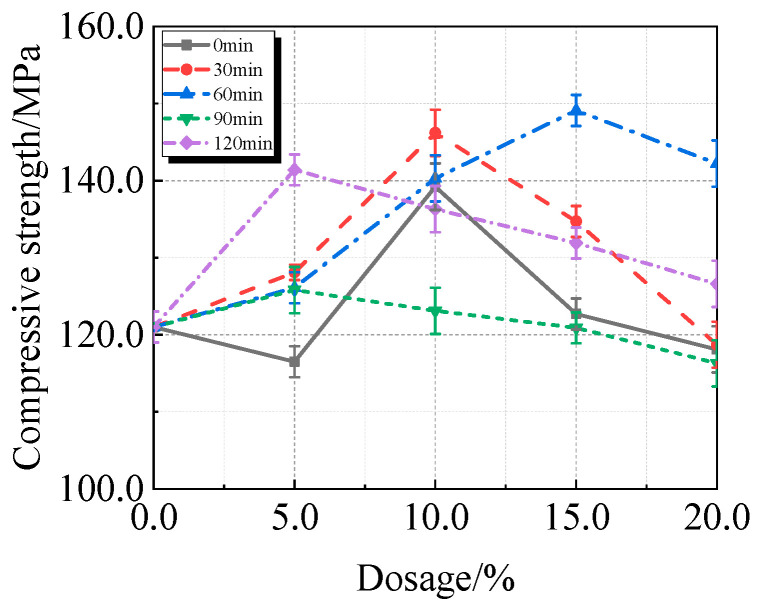
Compressive strength of UHPC mixed with calcined recycled fine powder ground for different durations.

The viscosity, fluidity, and compressive strength of UHPC comprising calcined recycled fine powder milled at various times are shown in Figure 7, Figure 8 and Figure 9. Figure 7 illustrates how the viscosity of UHPC progressively rises as the amount of calcined recycled fine powder increases. The UHPC, including calcined recycled fine powder ground for 120 min shows the greatest viscosity of 20,962.8 mPa·s when the content of calcined recycled fine powder reaches 20.0%. This is 353.5% greater than the reference UHPC without calcined recycled fine powder. The increased specific surface area and improved water absorption capacity of the ground recycled fine powder are responsible for the increase in viscosity of UHPC.

According to Figure 8, the fluidity of UHPC sharply declines between 15.0% and 20.0% of calcined recycled fine powder that is ground at the same time. As the grinding time increases, the fluidity of UHPC decreases with the same amount of calcined recycled fine powder. The decrease in UHPC fluidity is caused by both the longer grinding time and the higher concentration of calcined recycled fine powder. In comparison to the reference UHPC without calcined recycled fine powder, the UHPC containing 20.0% calcined recycled fine powder ground for 120 min exhibits a fluidity reduction of roughly 25.0%. The fundamental mechanism is that when recycled fine powder is calcined, hydration products such as AFt and Ca(OH)_2_ break down [25,26]. When these compounds come into contact with water, they rehydrate, which uses up mixing water. Additionally, the increased specific surface area of the particles from longer grinding times increases water demand, which raises the viscosity of UHPC and reduces its fluidity. The water can be consumed by the rehydration of these compounds when they come into contact with water. Additionally, the increased specific surface area of the particles due to the longer grinding time increases the water demand, which raises the viscosity and consequently decreases the fluidity of UHPC.

The compressive strength of UHPC is significantly increased by the addition of calcined recycled fine powder, as shown in Figure 9. A calcined recycled fine powder concentration of 15.0% and a grinding duration of 60 min yield the maximum compressive strength, reached 149.1 MPa, which is 23.2% greater than that of the reference UHPC without calcined recycled fine powder.

Following calcination, the recycled fine powder’s Si and Al structures disintegrate, changing from a stable to a metastable state [27]. By increasing the amount of cementitious materials in the hydration products and promoting the hydration reaction, synergistic interaction with volcanic ash and silicon fume [28]; Furthermore, the material particle size distribution is continuous, structural pores may be successfully filled, and the quartz sand is successfully contained in the cement–silica fume–calcined recycled fine powder ternary cementitious system [29], this transition improves the mechanical characteristics of UHPC that incorporates calcined recycled fine powder.

### 3.2. Effect of Activators on Properties of UHPC Mixed with Recycled Fine Powder

**Figure 10:**
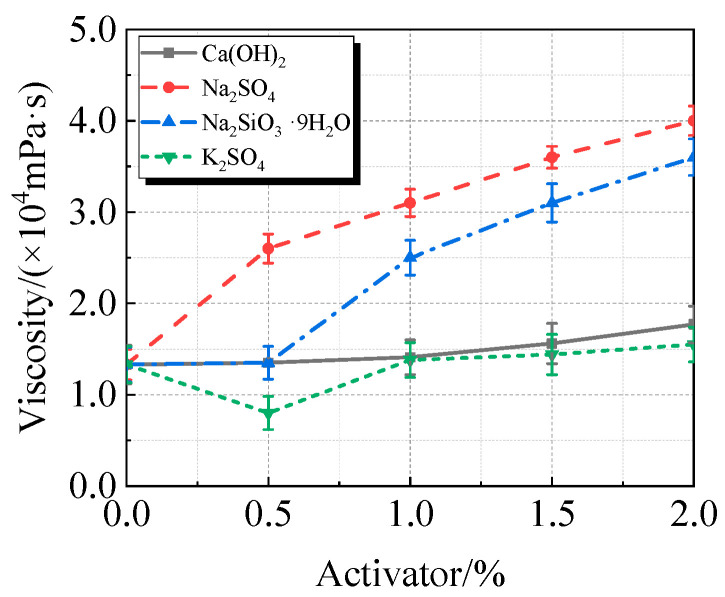
Viscosity of calcined recycled fine powder UHPC mixed with different dosages of activators.

**Figure 11:**
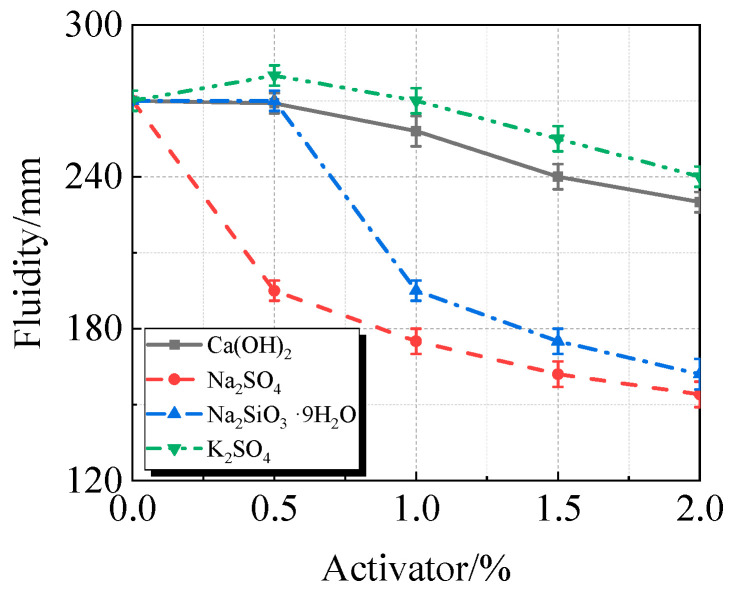
Fluidity of calcined recycled fine powder UHPC mixed with different dosages of activators.

**Figure 12:**
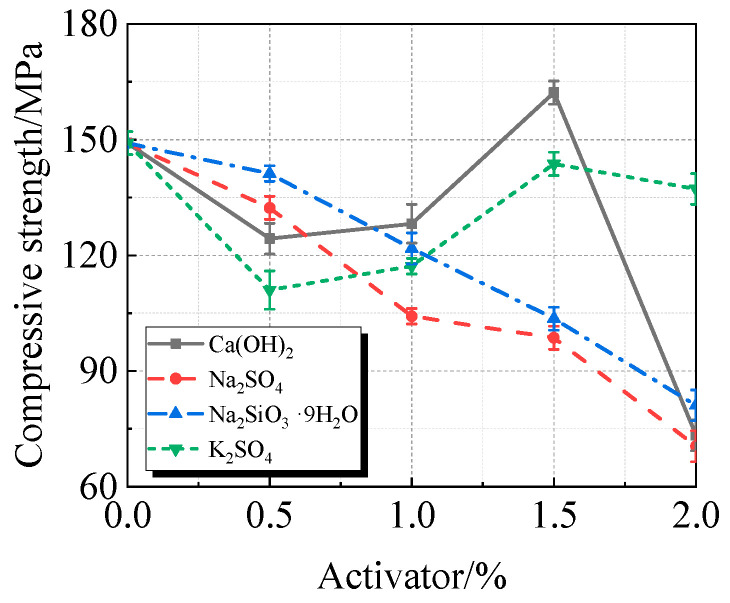
Compressive strength of calcined recycled fine powder UHPC mixed with different dosages of activators.

The viscosity, fluidity, and compressive strength of UHPC with varying concentrations of Ca(OH)_2_, Na_2_SO_4_, Na_2_SiO_3_·9H_2_O, or K_2_SO_4_, along with 15% calcined recycled fine powder milled for 60 min, are displayed in Figure 10, Figure 11 and Figure 12.

The addition of activators typically increases the viscosity and decreases the fluidity of UHPC containing calcined recycled fine powder, as seen in Figure 10 and Figure 11. At a 2.0% dosage, Na_2_SO_4_ had the most noticeable effect, increasing viscosity by 200.4% and decreasing fluidity by 43.0%. K_2_SO_4_, on the other hand, has the least impact on viscosity, increasing viscosity by 16.5% and decreasing fluidity by 11.1% at a dosage of 2.0%. In terms of viscosity and fluidity, the effects of Ca(OH)_2_ and Na_2_SiO_3_·9H_2_O fall between those of Na_2_SO_4_ and K_2_SO_4_.

Figure 12 illustrates how Ca(OH)_2_ significantly increases the compressive strength of UHPC that incorporates calcined recycled fine powder. When compared to the reference UHPC without any activator, the most significant improvement is seen at an ideal dosage of 1.5%, where the compressive strength reached 162.2 MPa, yielding an 8.8% increase in strength.

Ca(OH)_2_ enhances the UHPC’s alkaline environment using calcined recycled fine powder, encouraging cementitious material hydration. Concurrently, the Ca^2+^ takes part in the system’s ion exchange, resulting in the formation of C-S-H gel and other hydration products [30]. The mechanical property is further improved by these goods’ further development of an interwoven network structure.

### 3.3. Electrical Conductivity and pH Value

**Figure 13:**
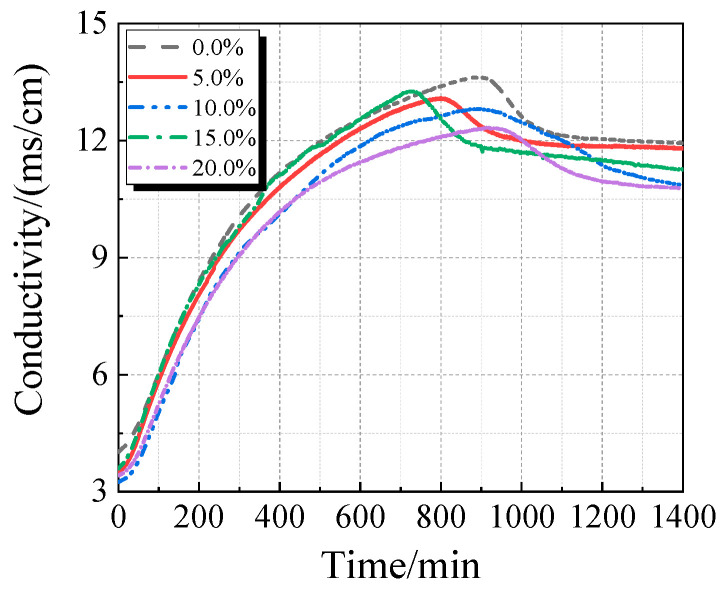
Conductivity of UHPC solution mixed with different contents of calcined recycled fine powder that was ground for 60 min.

**Figure 14:**
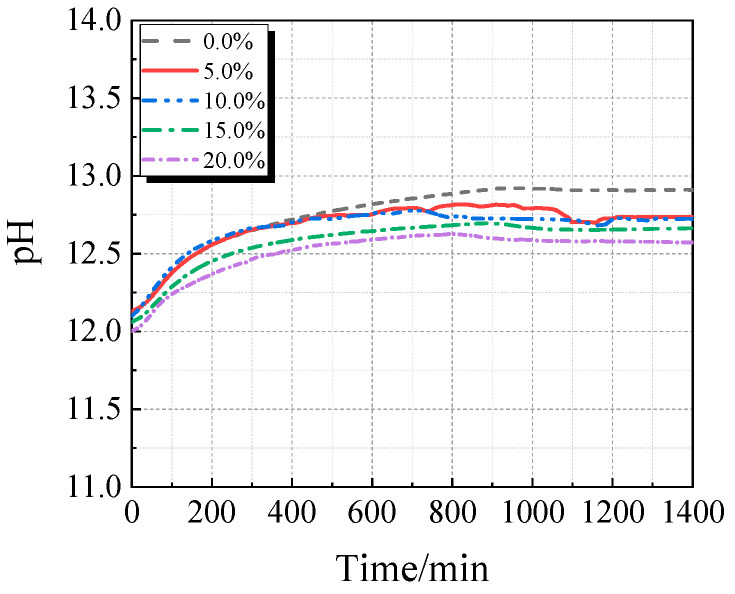
pH of UHPC solution mixed with different contents of calcined recycled fine powder that was ground for 60 min.

The electrical conductivity and pH of the UHPC solution with varying concentrations of calcined recycled fine powder crushed for 60 min are displayed in Figure 13 and Figure 14. As the dosage of the calcined recycled fine powder increases, the UHPC solution’s pH and electrical conductivity both drop, as Figure 13 and Figure 14 demonstrate. This pattern implies that the recycled fine powder’s ability to dissolve ions is lower than that of cement.

**Figure 15:**
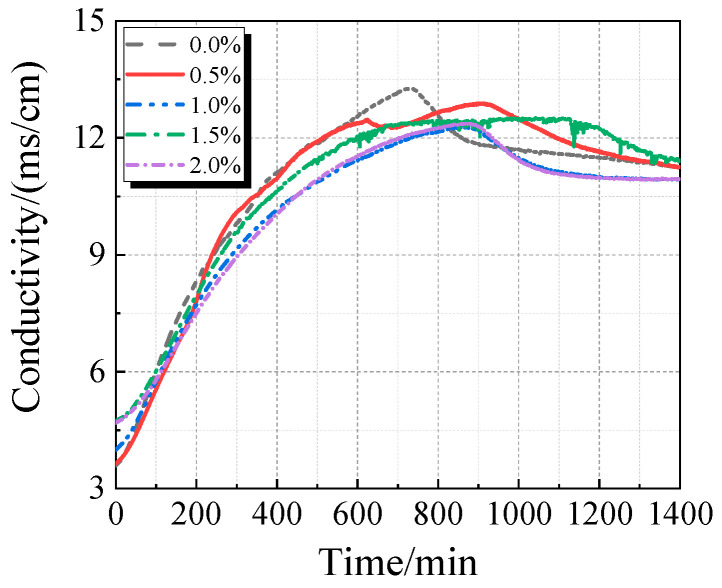
Conductivity of calcined recycled fine powder UHPC solution mixed with different dosages of Ca(OH)_2_.

**Figure 16:**
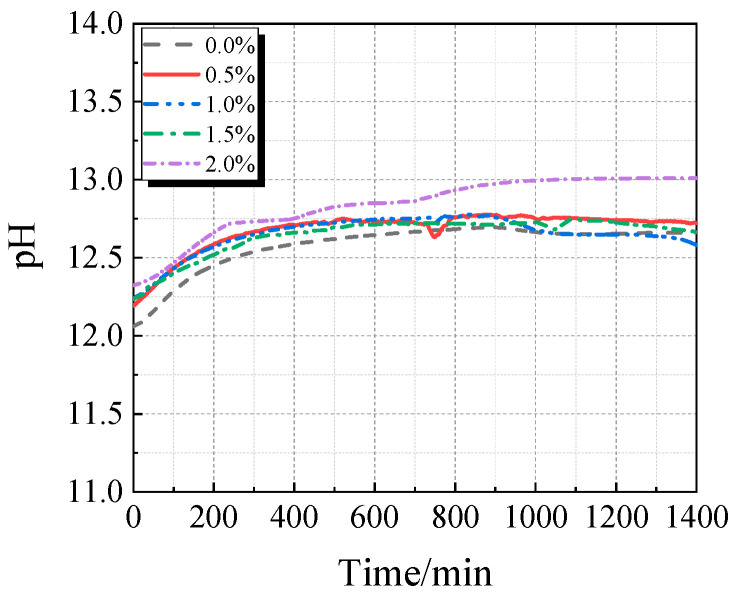
pH of calcined recycled fine powder UHPC solution mixed with different dosages of Ca(OH)_2_.

The electrical conductivity and pH value of the UHPC solution with calcined recycled fine powder and varying Ca(OH)_2_ dosages are displayed in Figure 15 and Figure 16. When Ca(OH)_2_ is added, the solution’s electrical conductivity drops, as seen in Figure 15. Ca^2+^ is released from the calcined recycled fine powder when it is combined with distilled water, increasing the electrical conductivity. But when Ca(OH)_2_ is added, the common-ion effect suppresses the dissolution of Ca^2+^ from the recycled powder [31], which lowers conductivity. Because Ca(OH)_2_ is naturally alkaline, as Figure 16 illustrates, the pH of the solution rises as the dosage of Ca(OH)_2_ increases.

### 3.4. XRD Analysis

**Figure 17:**
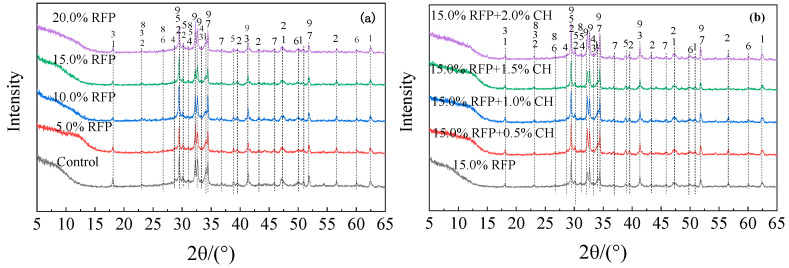
XRD pattern of hydrated UHPC specimen mixed with calcined recycled fine powder with and without of Ca(OH)_2_, (**a**) Recycled fine powder calcined and ground for 60 min, (**b**) Ca(OH)_2_ activated recycled fine powder. 1—Ca(OH)_2_ (Calcium hydroxide), 2—CaCO_3_ (Calcium carbonate), 3—Aft (Alunite), 4—CaSO_4_·2H_2_O (Gypsum), 5—C_2_S (Dicalcium silicate), 6—SiO_2_ (Quartz), 7—C_2_AS (Calcium alumina feldspar), 8—AFm, 9—C_3_S (Calcium silicate).

The XRD patterns of UHPC with varying amounts of calcined recycled fine powder crushed for 60 min with and without Ca(OH)_2_ are displayed in Figure 17. As the amount of calcined recycled fine powder increases, as seen in Figure 17a, the diffraction peaks of Ca(OH)_2_ and CaCO_3_ increase while those of C_3_S and C_2_S decrease. This suggests that the induction of calcined recycled powder can facilitate the hydration process. However, as the content of calcined recycled fine powder reaches 20.0%, the peaks of C_3_S and C_2_S become more prominent, suggesting a slowdown in the hydration rate. The high-water requirement of calcined recycled fine powder is the cause of this phenomenon. Because UHPC has a low water-to-binder ratio by nature, adding too much calcined recycled fine powder causes competition for water, which negatively impacts cement hydration. The addition of Ca(OH)_2_ weakens the diffraction peaks of C_3_S and C_2_S in the hydrated UHPC, as seen in Figure 17b, suggesting that Ca(OH)_2_ facilitates the hydration procedure. Nevertheless, the diffraction peaks of C_3_S and C_2_S rise when the Ca(OH)_2_ dose approaches 2.0%, indicating that an overly alkaline environment limits the hydration process [32], which ultimately results in a decrease in the compressive strength of UHPC. This is consistent with previous studies on the compressive strength patterns of UHPC with calcined recycled fine powder. The appropriate incorporation of calcined recycled fine powder helps the formation of hydration products, and the addition of Ca(OH)_2_ creates an alkaline environment conducive to hydration, further promoting the hydration process of the system, increasing the system’s pH, and enhancing the rate of ion dissolution.

### 3.5. TG-DTG Analysis

The breakdown of hydration products like C-S-H occurs in the first stage between 105 °C and 380 °C; the breakdown of Ca(OH)_2_ occurs in the second stage between 380 °C and 450 °C; and the breakdown of CaCO_3_ occurs in the third stage between 615 °C and 710 °C [33,34].

**Figure 18:**
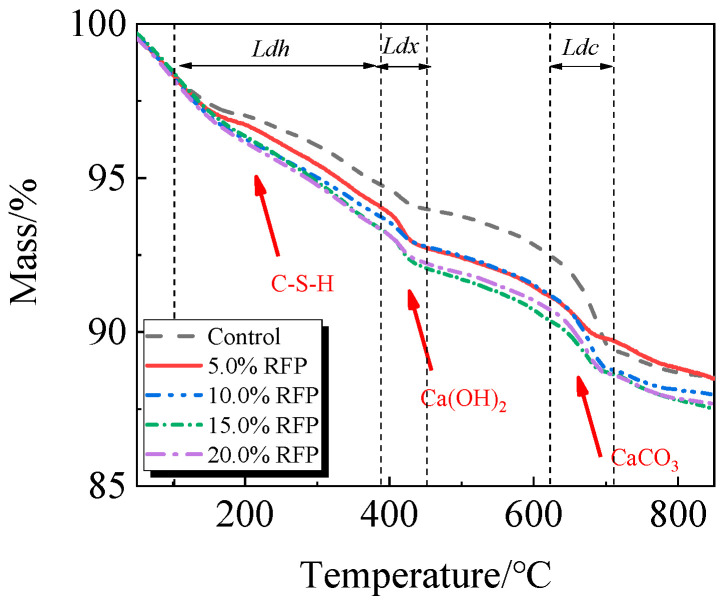
TG of hydrated UHPC mixed with calcined recycled fine powder.

**Figure 19:**
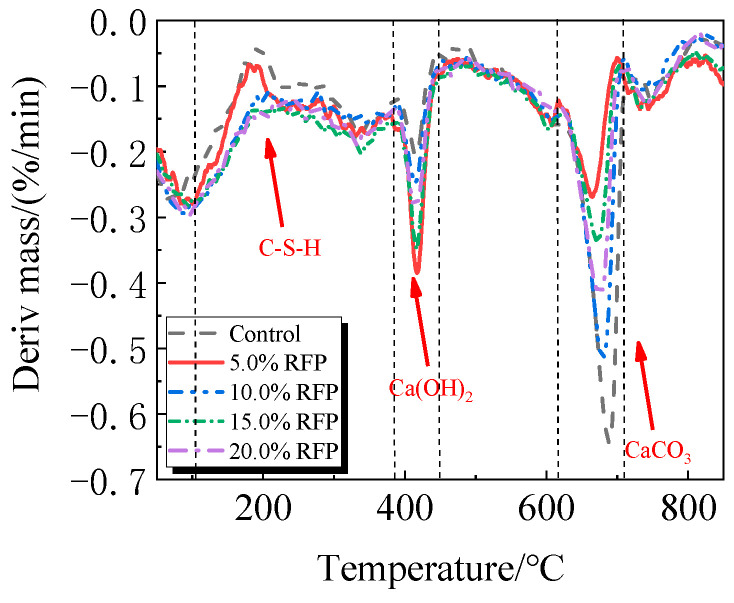
DTG of hydrated UHPC mixed with calcined recycled fine powder.

**Figure 20:**
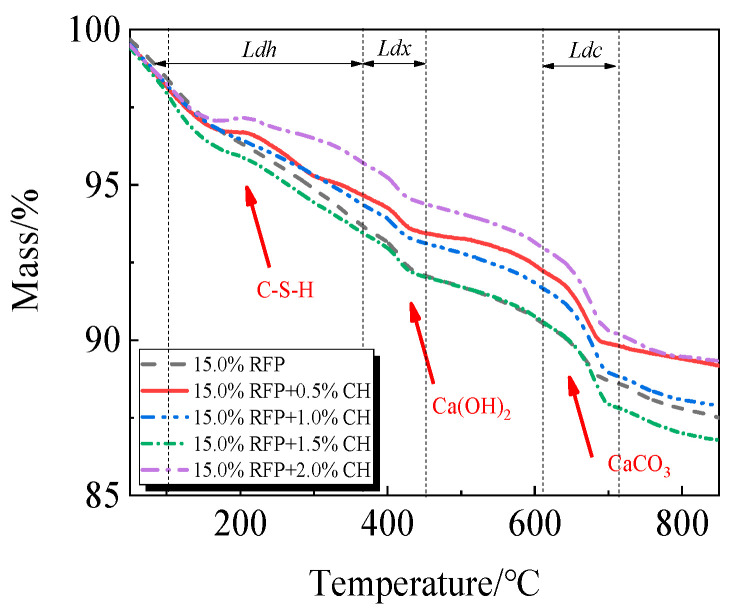
TG of UHPC mixed with different dosages of Ca(OH)_2_ and 15.0% calcined recycled fine powder.

**Figure 21:**
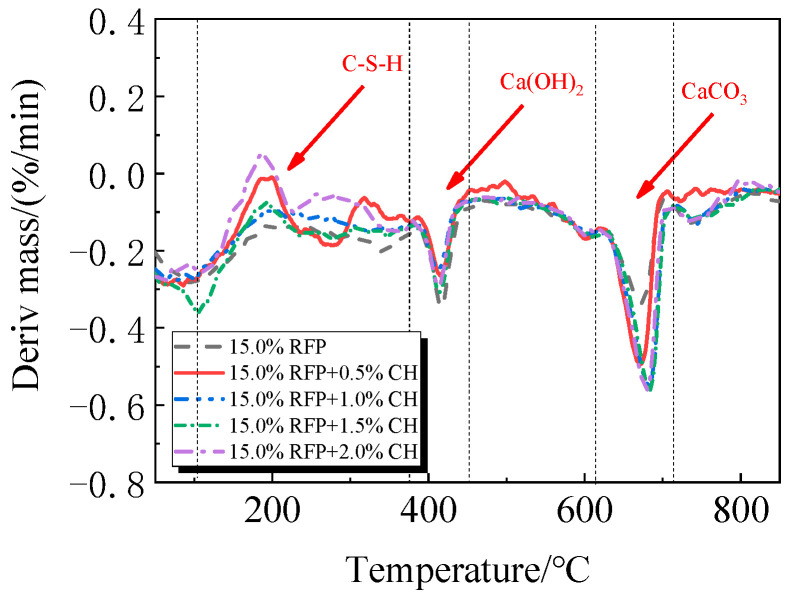
DTG of UHPC mixed with different dosages of Ca(OH)_2_ and 15.0% calcined recycled fine powder.

The TG-DTG of UHPC, including 0.0% to 20.0% calcined recycled fine powder milled for 60 min, is displayed in Figure 18 and Figure 19. Between 105 °C and 380 °C, the hydrated UHPC specimens containing calcined recycled fine powder show more mass loss than those without the powder, suggesting that the powder encourages the development of hydration products. In line with the findings in Figure 17a, the mass loss decreased when the dosage of calcined recycled fine powder surpassed 15.0%, indicating a reduction in hydration products. The TG-DTG curves of UHPC with 15.0% calcined recycled fine powder and 0.0% to 2.0% Ca(OH)_2_ are displayed in Figure 20 and Figure 21. The hydrated UHPC specimens show more mass loss between 105 °C and 380 °C at a Ca(OH)_2_ dosage of 1.5% than the specimens without Ca(OH)_2_ addition. It suggests that adding 1.5% Ca(OH)_2_ encourages UHPC hydration, resulting in the production of more hydration products. In line with the findings shown in Figure 17b, a decrease in mass loss was seen when the dosage of Ca(OH)_2_ exceeded 1.5%, suggesting a decrease in hydration products.

### 3.6. Degree of Hydration Analysis

Compressive strength and the degree of hydration of cement-based materials are favorably connected. Equations (1) and (2) can be used to determine the degree of hydration of UHPC based on the TG curve [35].

(1) $$W_{b}=Ldh+Ldx+0.41\;Ldc$$

(2) $$\alpha =W_{b}/0.24$$

where *W*_*b*_ and *α* represent the chemically bound water content and the degree of hydration of the specimen, respectively, and *Ldh*, *Ldx*, and *Ldc* represent the mass loss resulting from the breakdown of C-S-H gel, Ca(OH)_2_, and CaCO_3_, respectively.

**Figure 22:**
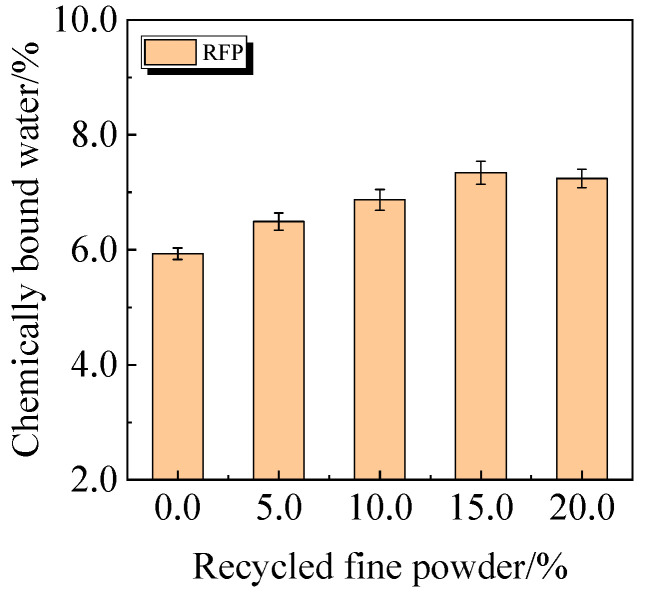
Chemically bound water of UHPC mixed with calcined recycled fine powder.

**Figure 23:**
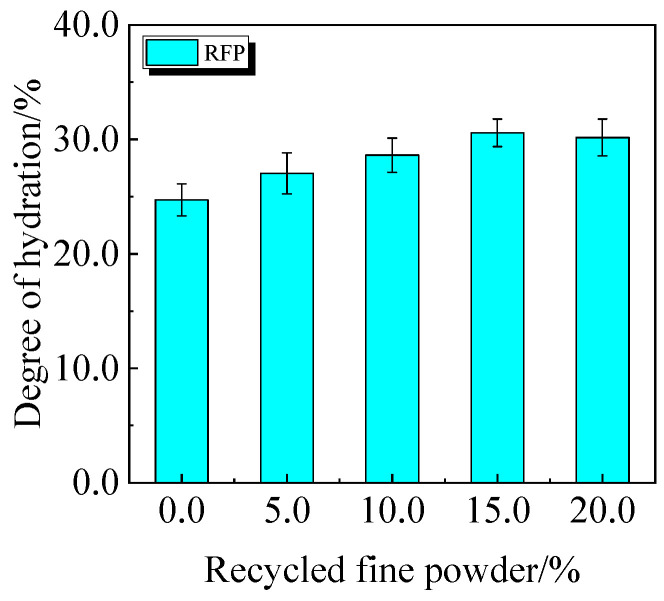
The hydration degree of UHPC mixed with calcined recycled fine powder.

The chemically bound water content and degree of hydration of UHPC, including 0.0% to 20.0% calcined recycled fine powder crushed for 60 min, are displayed in Figure 22 and Figure 23. According to the findings, a larger dosage of calcined recycled fine powder increases the amount of chemically bonded water and the degree of hydration when the dose ranges from 0.0% to 15.0%. Nevertheless, going over the 15.0% dosage has a negative impact on the hydration process.

**Figure 24:**
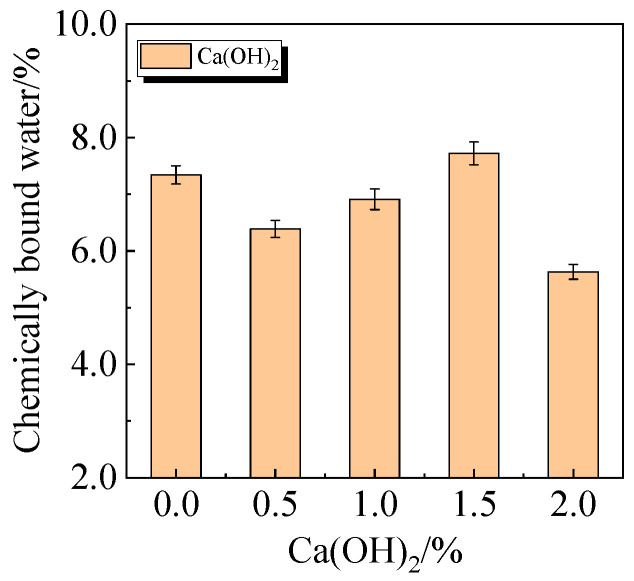
Chemically bound water of UHPC mixed with different dosages of Ca(OH)_2_ and 15.0% calcined recycled fine powder.

**Figure 25:**
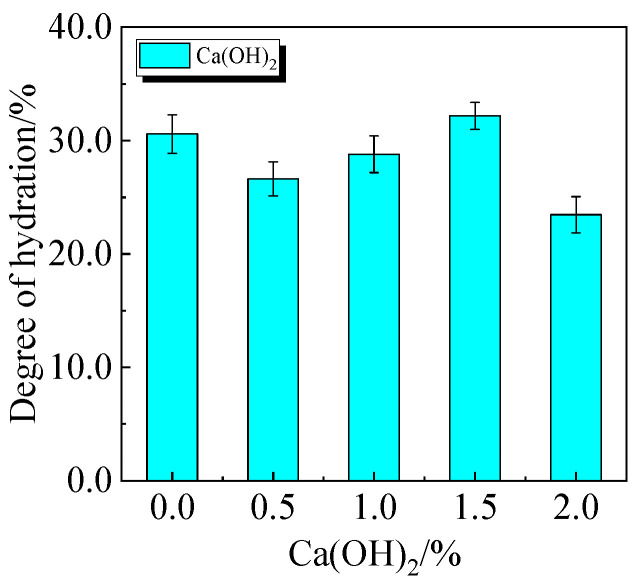
The hydration degree of UHPC mixed with different dosages of Ca(OH)_2_ and 15.0% calcined recycled fine powder.

The chemically bound water content and degree of hydration of UHPC, including 15.0% calcined recycled fine powder milled for 60 min with the addition of 0.0% to 2.0% Ca(OH)_2_, are displayed in Figure 24 and Figure 25. The hydration of the calcined recycled fine powder is much improved by the addition of 1.5% Ca(OH)_2_, as the figures demonstrate. The addition of calcined recycled fine powder and Ca(OH)_2_ improves the hydration process and the creation of hydration products, resulting in an overall increase in the degree of hydration. This investigation strongly supports the earlier XRD and compressive strength patterns.

### 3.7. SEM Analysis

**Figure 26:**
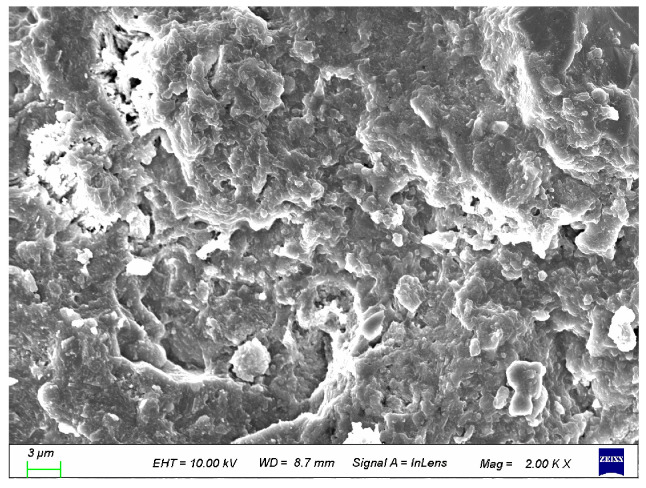
SEM of control group.

**Figure 27:**
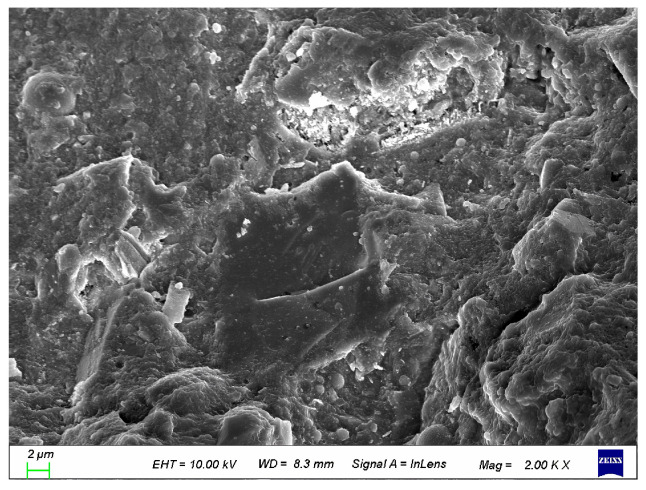
SEM of UHPC mixed with 2.0% of Ca(OH)_2_ and 15.0% calcined recycled fine powder.

To better analyze the structural impact of coupled activated recycled fine powder on UHPC, it was compared with the control group. As shown in Figure 26 and Figure 27, the incorporation of calcined recycled fine powder and calcium hydroxide makes the UHPC structure more compact. Therefore, it can be said that the coupled activated recycled fine powder has a significant enhancement effect on the hydration of UHPC, resulting in higher compressive strength [36].

### 3.8. Carbon Emission of UHPC Mixed Calcined Recycled Fine Powder

The carbon emission factors of the different raw materials utilized in UHPC are displayed in Table 3 [37]. Due to their low dose levels during UHPC synthesis and negligible effect on the overall carbon footprint, carbon emissions from chemical admixtures and activators are regarded as negligible.

The study’s recycled fine powder needed to be calcined and then ground. No appreciable CO_2_ emissions are produced since the calcination temperature was set at 600 °C, which is lower than the CaCO_3_ breakdown temperature. Electricity use is the main cause of carbon emissions during the calcination process. For high-temperature calcination, the carbon emission factor was 0.0622 kg·kg^−1^ [38], but for ball mill operation, it was 0.0161 kg·kg^−1^ [39]. As a result, 0.0973(0.0190 + 0.0161 + 0.0622) kg·kg^−1^ is the total carbon emission factor for the calcination and grinding of recycled fine powder. In comparison to the reference UHPC without the powder, UHPC with 15.0% calcined recycled fine powder shows a 12.9% decrease in carbon emissions, according to the data shown in Table 1 and Table 3. As a result, using calcined recycled fine powder in UHPC improves compressive strength and lowers carbon emissions, both of which support sustainable growth.

## 4. Conclusions

600 °C calcined recycled fine powder was ground and chemically activated to create ultra-high-performance concrete (UHPC). Analysis was performed on the calcined recycled fine powder grinding process, the choice of chemical activators, and their effectiveness. The following is a summary of the conclusions.

(1) The best qualities are shown by the recycled fine powder that was calcined at 600 °C for two hours and then milled for sixty minutes. UHPC shows a decrease in fluidity and an increase in viscosity when the dosage of the calcined recycled fine powder increases. UHPC reaches its maximum compressive strength at the ideal dosage of 15.0%, which is a 23.2% improvement over the reference UHPC without any recycled fine powder [40].
(2) The activators Ca(OH)_2_, Na_2_SO_4_, Na_2_SiO_3_·9H_2_O, and K_2_SO_4_ cause the UHPC containing calcined recycled fine powder to become more viscous and less fluid. Of them, Na_2_SO_4_ has the strongest effect and K_2_SO_4_ the least; the effects of Ca(OH)_2_ and Na_2_SiO_3_·9H_2_O are between those of K_2_SO_4_ and Na_2_SO_4_. Ca(OH)_2_ can efficiently boost the strength of UHPC; at a dosage of 1.5%, the maximum strength is reached, leading to an 8.8% increase in compressive strength above reference UHPC.
(3) UHPC hydration is enhanced by calcined recycled fine powder; nevertheless, the degree of hydration decreases when the dosage is above 15.0% [41]; The UHPC with 1.5% Ca(OH)_2_ shows the greatest amount of hydration products at the 15.0% dosage of calcined recycled fine powder; nevertheless, hydration is negatively impacted by dosages over 1.5% Ca(OH)_2_. Moreover, the carbon emissions linked to cement usage are successfully decreased by partially substituting calcined recycled fine powder for cement.

## Figures and Tables

**Table 1:** Basic mix proportion of UHPC (kg·m^−3^).

Material	Cement	Silica Fume	Quartz Sand		Water	Superplasticizer
			425–212 μm	212–106 μm		
Mix proportion/(kg·m^−3^)	1330.8	190.1	456.3	456.3	273.8	16.7

**Table 2:** D_10_, D_50_, and D_90_ of calcined recycled fine powder ground for different times.

No.	Calcination Temperature/°C	Calcination Time/h	Grinding Time/min	D_10_/μm	D_50_/μm	D_90_/μm
1	600	2	0	16.64	148.00	403.70
2	600	2	30	4.33	43.64	174.10
3	600	2	60	3.81	39.22	144.50
4	600	2	90	3.19	29.83	110.80
5	600	2	120	2.80	24.02	93.86

**Table 3:** Carbon emission factors of raw materials for UHPC (kg·kg^−1^).

Material	Cement	Silica Fume	Quartz Sand	Recycled Fine Powder
Carbon emission factors/(kg·kg^−1^)	0.7590	0.0240	0.0102	0.0190

## Data Availability

The original contributions presented in this study are included in the article. Further inquiries can be directed to the corresponding author.
